# Chronic disease knowledge and its determinants among chronically ill adults in rural areas of Shanxi Province in China: a cross-sectional study

**DOI:** 10.1186/1471-2458-11-948

**Published:** 2011-12-22

**Authors:** Miaomiao Tian, Yingchun Chen, Rui Zhao, Li Chen, Xi Chen, Da Feng, Zhanchun Feng

**Affiliations:** 1 School of Medicine and Health Management, Huazhong University of Science and Technology, Wuhan, Hang Kong Road, China

## Abstract

**Background:**

Chronic disease knowledge is an important prerequisite for an individual to implement behavioural changes towards the prevention and control of chronic diseases (CDs). Limited information is available about the relationship between different levels of health services and CD knowledge among rural residents with CDs. This research explores the distribution characteristics of CD knowledge and its determinants among chronically ill adults in rural China according to the aspects of patients and health service providers.

**Methods:**

A cross-sectional study was undertaken to estimate distribution characteristics of CD knowledge and collect data of socio-demographic characteristics, healthcare institutions attendances, duration of illness, and family history of CDs. Participants were 1060 rural adults with hypertension or type II diabetes. Correct responses to 12 questions were summed into a total knowledge score, and participants were divided into an adequate health knowledge group (score ≥ 6) or an inadequate health knowledge group (score < 5). Logistic regression was used determine the predictors of adequate CD health knowledge.

**Results:**

The mean age of participants was 61.34 years (SD = 10 years). Out of a possible 12, the median score on the CD knowledge questionnaire was 3.0. About 25% of participants were classified as having adequate CD knowledge. Those who had a family history and/or long duration of CDs were more likely to have adequate health knowledge. Participants who received CD health information and self-care instructions from their physicians had 2.67 and 13.34 times greater odds of possessing adequate health knowledge than those who received no information, respectively. Adequate CD knowledge was strongly associated with regular check-ups, especially for those who attended township hospitals (OR = 40.17).

**Conclusions:**

Having regular check-ups at a fixed healthcare institution and receiving health information from physicians are important measures for increasing CD knowledge among rural adults with CDs. Township hospitals are the most effective settings for health education. It is important to develop an effective community-based prevention and control mechanism for CDs. This requires township hospitals to take a leading role in improving CD knowledge among chronically ill patients, and enhancing implementation of health education in rural China.

## Background

Chronic diseases (CDs) are the leading global causes of death, and they are concentrated in the world's low- and middle-income populations. CDs were responsible for 36 million of the 57 million deaths that occurred globally in 2008, and mainly comprised cardiovascular diseases, cancers, and diabetes. Nearly 80% of CD deaths occur in low- and middle-income countries [1]. In 2008, data of the Fourth Chinese National Health Services Survey showed that 270 million Chinese people were diagnosed with at least one chronic disease, and about 82% of deaths and 70% of disability-adjusted life years lost were caused by CDs. By 2020, it is predicted that the number of deaths attributable to CDs will rise to 85% [2]. The rapidly growing burden of chronic illnesses in low- and middle-income countries threatens the health condition of their population and their economic development. Because most health-care costs must be paid by patients out of their own income in these countries, the cost of health care for CDs creates significant strain on household budgets. Each year, an estimated 100 million people are pushed into poverty because they have to pay directly for health services [1,3]. In China, indirect economic loss caused by CDs was 4.1% of GNP in 2000, and rural residents have taken 65% more economical risk of CDs than urban residents. Rural people had greater likelihood of impoverishment cause of treatment of CDs [4].

CDs are diseases of long duration and generally slow progression that are largely preventable through the reduction of their main lifestyle risk factors, such as unhealthy diet, physical inactivity, tobacco use, and harmful use of alcohol [5]. Research shows that increasing the health knowledge of chronically ill and high-risk populations is the most effective way to prevent and delay the onset of CDs [6,7]. The improvement of health knowledge contributes to an increased awareness of health care, which not only helps people know more about diseases and increases their ability for self-care, but also helps them to quit unhealthy behaviours and lead healthy lifestyles. In rural China, health care providers are the main disseminators of health knowledge. Compared with urban residents, in 2008, the rural population had a lower educational level with 18.9% of them illiterate and 69.2% not having attended senior middle school. Accessing the rural population for the purpose of providing health information is difficult. Most of them obtain health knowledge mainly from the television and their physicians, but there is no systematic television broadcast of health information [2]. Therefore, the most effective way to improve rural residents' health knowledge and encourage health behaviour is through heath education and promotion.

Trying to prevent, control, and curb the growth trend of CDs in rural areas is a main activity of the Ministry of Health of China. More than half of the Chinese population (53.41% of 1.3 billion in 2009 [8]), still live in rural areas where residents cannot access the same level of health services as their urban counterparts, which is partly because of a lower level of economic growth, lack of amenities, and poor health service capacity in rural areas [9]. In 2008, the prevalence of CDs in rural China was 17.1%, a 5% increase on the 2003 prevalence, which is a faster rate of increase than that observed in the urban population (24% in 2003, 28.3% in 2008) [10]. Shanxi Province is an example of a large middle-income province of China that is located in north China. It has a population of 33.92 million, of which 55.97% live in rural areas. Heart attack, cancer, stroke, diabetes, and chronic obstructive pulmonary disease are now the leading causes of death in rural areas among middle-aged and elderly people, and represent a major burden on CD prevention and control.

In China, county hospitals, town hospitals, and village clinics compose the three-tier rural health service network. They are the principal providers of health care services to rural residents to maintain their health. They are also responsible for CD prevention and control. Compared with countries that implement a family physician practice system of primary health care [11], Chinese citizens can choose to have their check-ups at any hospital (i.e. village clinic, community health services centre, municipal hospital, or provincial hospital). It was reported that 81.7% of rural residents preferred to obtain health care services from village clinics and town hospitals [12]. Therefore, primary health care institutions are the major 'health keepers' for rural residents. Research shows that the health service capabilities of health institutions, to some extent, determines the health level of local residents; which is influenced by disease treatment, public health services, and health promotion and education, with the latter having an enormous influence on people's level of health knowledge [13,14].

So far, most investigations of the factors contributing to CD-related knowledge among chronically ill adults mainly focused on the characteristics of patients instead of health providers. It has been demonstrated that educational level, occupation, rural/urban statuses of residence, and region of residence were risk factors for inadequate or low CD knowledge [15-19]. American researchers have confirmed that the receipt of health knowledge from different health care providers (practitioners, nurses, diabetes educators, physicians or pharmacists) was a key contributor to one's health knowledge [20]. Yet, to our knowledge, there are no studies analysing the influence of health institution services on the health knowledge of chronically ill adults in China. Therefore, we set out to assess the determinants of CD knowledge from the perspectives of both patients and health providers, and analyse the influence of health care services (of the three-tier rural health institutions) on the health knowledge of rural residents with CDs. Specifically, our research questions are: (1) How is CD knowledge distributed among chronically ill rural residents? (2) What are the potentially modifiable factors associated with CD knowledge? (3) What is the influence of different health institutions on CD knowledge among rural adults with CDs? A cross-sectional survey was used to answer these questions.

## Methods

### Study population

The data were collected using a cross-sectional survey conducted in July 2009. We chose Shanxi Province as the study site because chronic disease screening was undertaken in this area during March-June of 2009 [21]. The province consists of 11 cities with 85 counties, and 23 county-level cities that are geographically divided into 2 strata, as urban, or rural regions. Using a stratified multi-stage sampling method, 4 cities in the urban region, 4 counties, 12 townships, and 36 villages in the rural region were selected following the sequence of district-block-residential area. We selected 30 chronically ill adults from each village from the eligible candidates listed in health records, which were divided into three groups according to the distance from home to the village clinic, and 10 samples were randomly selected from each group. The eligible candidates were defined as those who had been diagnosed with hypertension or type II diabetes (T2D) by physicians, and aged older than 18 years. A total of 1060 rural residents with hypertension or T2D aged from 30-80 years were randomly sampled from 36 villages, and all participants completed a CD survey, including a section on health knowledge. The majority of participants (874) were diagnosed with hypertension, and 186 participants were diagnosed with T2D.

All participants were interviewed in person, and the study purpose was explained to them by the interviewers. Students from the School of Medicine and Health Management of Tongji Medical College and staff of local health care institutions were recruited and trained as interviewers. Items and response choices were read to participants who had difficulty reading (because of illiteracy or poor vision).

This study was approved by the Ethics Committee of Tongji Medical College, Huazhong University of Science and Technology (IORG No: IORG0003571) and informed consent was obtained from all participants in the study.

### Questionnaire

The questionnaire was delivered in simplified Chinese and contained a combination of open- and closed-ended questions (true/false format). It consisted of three parts: socio-demographic information, CD health knowledge, and the ways of receiving health knowledge. Participants were also asked questions about their duration of illness (hypertension/T2D), family history of CDs, and the health institutions they attended for regular check-ups (usually once a month) (Additional file 1).

Socio-demographic information collected included sex, age, educational level, marital status, and employment status.

Health knowledge items were developed by referring to the National Health Promotion Project for Hundreds of Millions of Chinese Farmers (NAHPF) [21], which was launched by the Ministry of Health of China in 2006. We selected all items about CDs from the NAHPF item pool and classified them into 3 groups: basic knowledge (including normal adult blood pressure range for participants with hypertension; normal adult glucose range for participants with diabetes), the risk factors for CDs (6 questions), and daily self-care techniques (5 questions). With the exception of the basic knowledge questions, which had an open response, the response options were: "true", "false", and "don't know".

Sources of health information included the participant's means of receiving health knowledge, and the fixed health institution that participants attended for regular check-ups.

### Statistical methods

All data were entered in duplicate into the EpiData Info version 3.1 database and statistics program (Atlanta, Georgia, USA), and data entry screens were used to revise incorrect entries (i.e. logic errors, input errors). Statistical analysis was performed using PASW statistics 18.0 (SPSS, Chicago, IL, USA).

The overall CD knowledge score was determined based on the sum of correct answers to 12 health knowledge questions. Each correct answer received one point. Incorrect, missing answers, or the answer "don't know" were awarded no points. According to the goal of NAHPF in 2010 [22] and the level of CD-related health knowledge among rural residents in Shandong (about 35% in 2007) and Anhui province (about 37% in 2008), which had a similar GDP to Shanxi province, we defined the cut-off score for adequate versus inadequate knowledge as 6 points (more than 50% correct answers) [23,24]. Those at/or above the cut-off score (6 or more items answered correctly) were considered to have adequate health knowledge, and those below it (5 or fewer correct responses) we considered to have inadequate knowledge.

Socio-demographic characteristics, duration of illness (hypertension/T2D), means of receiving health information, CD health knowledge, and regularly-attended health institutions were summarized using descriptive statistics. Prior to developing logistic regression analysis of the predictors of CD health knowledge, Chi-square and Fisher's exact tests (where appropriate) were used to explore differences in values of the covariates between the adequate and inadequate health knowledge groups for the purpose of selecting meaningful variables for regression (p-value < 0.05). Binary logistic regression analysis was used to model the probability of participants having adequate CD health knowledge. The dependent variable was whether (1) or not (0) each participant had adequate CD health knowledge. Independent variables included: sex, age, educational level, duration of illness (hypertension/T2D), family history of CDs, regular attendance at health institutions, and whether health information and self-care instruction was received. All reported p-values are two-sided, and statistical significance level (α) was set at 0.05.

## Results

### Socio-demographic characteristics of participants

Participants were predominantly middle-aged or older and had a mean age of 61.34 years (SD = 10.04 years, range 30-90 years) with the majority being female (67.9%). More than 70% of participants were at/or below elementary educational level. Nearly one third of them (32.74%) had a family history of CDs (hypertension or diabetes). Other socio-demographic information is presented in Table 1.

**Table 1:** Socio-demographic characteristics of rural adults with CDs in Shanxi Province of China

Characteristics	Participants (n = 1060)	Percent (%)
**Gender**		

Male	340	32.10

Female	720	67.90

**Age**		

30-39	19	1.79

40-49	101	9.53

50-59	311	29.34

60-69	386	36.42

70-	243	22.92

**Education level**		

less than 6 years elementary study	398	37.50

Elementary	365	34.40

Middle school	236	22.30

high school and above	61	5.80

**Chronic diseases**		

Hypertension	874	82.50

Diabetes	186	17.50

**Marital Status**		

Unmarried	11	1.00

Married	878	82.80

Divorced	7	0.70

Widowed	164	15.50

**Occupation**		

Farmer	724	68.30

migrant worker ^a^	19	1.80

self-employed ^b^	15	1.40

factory workers	90	8.50

no work	212	20.00

**Family history of chronic diseases**		

Yes	347	32.74

no	713	67.26

**Length of illness(hypertension/T2D)**		

1-5	523	49.34

6-10	316	29.81

11-15	118	11.13

16-	103	9.72

Note: a. migrant worker means the rural people leave their villages and go into the cities to do an off-farm work. b. self-employment labourers refer to people who run a private small-scale business by themselves, such as vendors, food-shop owners, repair-shop owners and rice-noodle sellers

### CD health knowledge

Out of a possible score of 12, the mean and standard deviation of number of correct answers was 3.90 and 3.21 respectively. The median number of items answered correctly was three. Seventeen participants (1.6%) responded correctly to all health knowledge questions. The number of respondents who had adequate CD health knowledge (i.e. six or more correct items) was 266 (25.1%). The mean number of correct items' of the adequate knowledge group was 8.56, and that of the inadequate knowledge group was 2.34.

Slightly less than 40% of rural participants with hypertension knew the normal blood pressure range of healthy adults, and only 11.8% of participants with diabetes knew the normal adult glucose range. Two thirds of participants (77.26%) knew that patients with CDs could control their diseases by following their physicians' medical orders. However, only 15.28% recognized that losing weight was a normal way to control CDs.

### The ways of receiving health knowledge

The main ways that rural participants received CD health knowledge were from physicians, television programs, family members, or neighbours (81.32%, 17.64%, 16.98% and 12.92%, respectively). Of the 75.00% of participants who wanted to share their CD knowledge with other people, 95.85% wanted to share it with family members, 77.99% with their neighbours and 55.73% with other chronically ill people. About 12.83% of participants would like to spread CD knowledge to all people in the region. Overall, physicians were the main sources of health knowledge, and chronically ill adults are willing disseminators of CD knowledge. Health information about CDs (i.e. risk factors, and how long to measure blood pressure or blood glucose) from physicians was received by 60.1% of participants, and 763 reported that physicians had given them self-care instructions (i.e. stop smoking, do physical exercise, diet control) for daily life.

Regular receipt of health care services at a fixed health institution (usually once a month) was reported by 860 rural chronically ill adults. Among them, 54.77% saw a physician at village clinics, 30.81% attended township hospitals, and 14.42% attended county hospitals. However, the distribution of participants across the three types of health care providers was different for each of the health knowledge groups. Of the participants with adequate health knowledge, 73.3% attended township hospitals, which was far more than people with inadequate health knowledge (8.8%). Among the inadequate health knowledge group, more than half of the rural chronically ill adults (55.9%) attended village clinics, and 24.9% of these participants did not have regular check-ups at fixed medical institutions. There was a significant difference between the two groups distribution across the health care providers, tested by a chi-square test with *p *< 0.01.

### Factors affecting CD health knowledge among adults with hypertension or T2D

Results of univariate analyses indicated that socio-demographic characteristics including age, marital status, education level, and duration of illness had significant relationships with adequate CD health knowledge (all p-values < 0.05). Regular healthcare from fixed health institutions, and receipt of CD-related health and self-care information from physicians were also correlated with CD health knowledge (all p-values < 0.001) (Table 2).

**Table 2:** Univariate analyses examining factors associated with adequate health knowledge in rural chronically ill adults

Predictor	Group of adequate health knowledge (n = 266)		Group of inadequate health knowledge (n = 794)		χ2	P
	
	N	%	N	%		
**Sex**						

male	92	34.6	248	31.2		

female	174	65.4	546	68.8	1.028	0.311

**Age**						

< 50	30	11.3	90	11.3		

50-59	84	31.6	227	28.6		

60-69	113	42.5	273	34.4		

70-	39	14.7	204	25.7	15.791	**0.003**

**Marital Status**						

Unmarried	4	1.5	7	0.9		

Married	234	88	644	81.1		

Divorced	2	0.8	5	0.6		

Widowed	26	9.8	138	17.4	9.371	**0.025**

**Education level**						

less than 6 years elementary study	80	30.1	318	40.1		

Elementary	92	34.6	273	34.4		

Middle school	72	27.1	164	20.7		

beyond high school	22	8.3	39	4.9	12.869	**0.005**

**Duration of illness**						

1-5	114	42.9	403	50.8		

6-10	81	30.5	235	29.6		

11-15	46	17.3	78	9.8		

16-	25	9.4	78	9.8	12.138	**0.007**

**The place participants received health care regularly**						

Village Clinics	27	10.2	444	55.9		

township hospitals	195	73.3	70	8.8		

County Hospitals	42	15.8	82	10.3		

participants who do not check up regularly in clinics or hospitals	2	0.8	198	24.9	492.274	**< 0.001**

**Family history of chronic diseases**						

yes	138	51.9	260	32.7		

no	128	48.1	534	67.3	31.109	**< 0.001**

**Received health information related to chronic diseases from doctors**						

yes	252	94.7	385	48.5		

no	14	5.3	409	51.5	177.712	**< 0.001**

**Received self-care instructions** **from doctors**						

yes	263	98.9	500	63		

no	3	1.1	294	37	127.325	**< 0.001**

Note: adequate health knowledge of CDs = answer at and more than 6 items correctly

With the exception of sex, all variables were tested for association with CD health knowledge status using multivariate logistic regression. Results indicated that age was the only socio-demographic variable that was significantly associated with CD health knowledge, with older people being more likely to have adequate knowledge. With increasing duration of CD, participants were more likely to have adequate health knowledge. Meanwhile, rural residents who had a family history of CDs had 1.97 times the odds of having adequate health knowledge than those with no family history (OR = 1.966, 95% CI = 1.311-3.04, *p *= 0.001) (Table 3).

**Table 3:** Multivariable analyses examining factors associated with adequate health knowledge in rural chronically ill adults

Predictor	Reference category	B	P	OR	95% CI
**Age**	< 50				

50-59		0.491	0.159	1.633	(0.825, 3.232)

60-69		0.981	**0.006**	2.667	(1.326, 5.366)

70-		0.422	0.337	1.525	(0.645, 3.606)

**Marital Status**	Unmarried				

Married		0.416	0.631	1.515	(0.278, 8.245)

Divorced		0.470	0.767	1.601	(0.071, 36.010)

Widowed		0.269	0.770	1.309	(0.215, 7.958)

**Education level**	Less than 6 years elementary study				

Elementary		-0.425	0.110	0.654	(0.389, 1.101)

Middle school		0.057	0.847	1.059	(0.591, 1.898)

Beyond high school		0.032	0.942	1.033	(0.433, 2.463)

**Duration of illness**	1-5				

6-10		-0.102	0.679	0.903	(0.558, 1.463)

11-		0.697	**0.013**	2.007	(1.161, 3.469)

**Family history of chronic diseases**	no	0.691	**0.001**	1.966	(1.311, 3.041)

**The place participants received health care regularly**	Participants who do not check up regularly in clinics or hospitals				

Village Clinics		0.148	0.851	1.159	(0.249, 5.384)

Township hospitals		3.693	**0.000**	40.173	(8.646, 186.654)

County Hospitals		1.889	**0.018**	6.616	(1.383, 31.644)

**Received health information related to chronic disease**	Have not received health information related to chronic diseases	0.982	**0.006**	2.671	(1.333, 5.350)

**Received self-care instruction from doctors**	Have not received self-care instruction from doctors	2.591	**0.000**	13.340	(3.634, 48.972)

Note: adequate health knowledge of CDs = answer at and more than 6 items correctly

Having regular check-ups in a fixed health care institution was positively associated with adequate CD health knowledge. Among the three-tiers of health care institutions, participants that attended township hospitals as their fixed healthcare institution had nearly 40 times the odds of having adequate health knowledge than those who did not see a physician regularly (OR = 40.173, 95% CI = 8.646-186.654, *p *< 0.001). Those who attended county hospitals 6.62 times greater odds (OR = 6.616, 95% CI = 1.383-31.644, *p *= 0.018) of having adequate health knowledge compared with those who did not see a physician regularly. Interestingly, participants attending village clinics showed no significant difference in likelihood to have adequate health knowledge than those who did not see a physician regularly (OR = 1.159, 95% CI = 0.249-5.384, *p *= 0.851). Participants who had received CD health information from their physicians had 2.67 times greater odds of having adequate health knowledge than those did not receive CD health information (OR = 2.671, 95% CI = 1.333-5.350, *p *< 0.001). Also, participants that received self-care instructions from physicians had 13.34 times greater odds of having adequate CD knowledge than those who received no instructions (OR = 13.340, 95% CI = 3.634-48.972, *p *< 0.001) (Table 3).

### Association between receiving health information and CD health knowledge

The type of health care institution regularly attended by chronically ill rural residents, and the receipt of health information and self-care instructions from physicians had a strong joint effect on CD health knowledge (Table 4). Among 265 participants who had regular check-ups in township hospitals, 249 (93.96%) self-reported that they had received health information from physicians, and 255 (96.23%) received self-care instructions, and about 76% of them belonged to the adequate CD health knowledge group. However, among 471 participants who had regular check-ups in village clinics, 233 (49.47%) had received health information from their physicians (8.15% of them scored ≥ 6) and 372 (79%) received self-care instructions (7% of them scored ≥ 6).

**Table 4:** Association between health institutions and CD health knowledge

Health institutions	Participants	Received health information related to CDs from doctors		Group1*		Group2**		Received self-care instructions from doctors		Group1*		Group2**	
	**N**	**N**	**%**	**N**	**%**	**N**	**%**	**N**	**%**	**N**	**%**	**N**	**%**

**Village clinics**	471	233	49.47	19	8.150	214	91.85	372	78.98	26	6.99	346	93.01

**Township hospitals**	265	249	93.96	191	76.71	58	23.29	255	96.23	195	76.47	60	23.53

**County hospitals**	124	109	87.90	42	38.53	67	61.47	105	84.68	41	39.05	64	60.59

**No fixed health institutions^a^**	200	46	23.00	0	0	46	100	31	15.50	1	3.23	30	96.77

Note: a. no fixed health institutions mean that participants do not see a doctor regularly in a fixed health institution. * the group of participants having adequate health knowledge of CDs. ** the group of participants having inadequate health knowledge of CD

The proportions of participants who received health information and self-care instructions from county hospitals were around 86%, and about 39% of them were classified as having adequate CD health knowledge. Those who had not attended regular check-ups in a fixed health institution had the lowest potential to receive health knowledge from physicians, and were thus less likely to have adequate CD health knowledge.

## Discussion

More than 70% of participants in our study had inadequate CD heath knowledge, including the highly educated rural participants who had high school education or higher. Participants had a higher level of basic CD knowledge than professional knowledge. For example, they know about the normal blood pressure range of healthy adults, and disease control through smoking cessation and reasonable alcohol consumption, but few of them know about hyperglycaemia and hyperlipidaemia as risk factors for hypertension (with only 17.36% of them answering correctly), and that weight loss is a means of disease control (with just 15% answering correctly). These finding are consistent with studies conducted in other provinces of China [24-26].

As is indicated in the multivariate model, participants who had a longer duration of CDs, and those who had a family history of CDs were more likely to have adequate CD health knowledge, which may have led to increased attention on disease control for themselves and their family members over time. In such circumstances, they may have been more willing to see physicians and obtain health information and self-care instructions from them than people without a family history of CD, or with a short duration of CD.

Our findings convince us that those who receive health information and self-care instructions from their physicians are more likely to have adequate CD health knowledge. The information in Table 3 reveals that adequate CD health knowledge among the respondents was associated with receiving health care from regularly-attended fixed health institutions. We also identified a significant relationship between the health services of different health institutions and the CD knowledge of participants who had regular check-ups.

As part of the Chinese Health System Reform (initiated in 2009), CD management and health education are main components of basic public health services, and are at the top of the policy agenda for rural health [27]. The Ministry of Health has outlined the main responsibilities of each level of health institution for CD prevention and control. Village clinics, the basic part of the rural health care system, are responsible for providing basic outpatient diagnosis and treatment, basic public health care including health education and medication management, and long-term monitoring for patients with CDs. In addition to supplying health care services to patients with CDs, township hospitals are also obligated to help to build and maintain health records, conduct health education and promotion, and supply service guidance and technical training for village clinics. County hospitals, as the medical centre for a county area, also have a responsibility to provide service and technical guidance for township hospitals and village clinics.

According to our findings, township hospitals have the most positive influence on CD knowledge for rural chronically ill residents among the three-tier rural health care institutions. Almost all participants in township hospitals (93.96%) received health education and promotion services; and most of them had significantly higher scores of CD knowledge. While more than half of the participants attending village clinics self-reported that they had received such services from village physicians, less than 10% of them had adequate CD health knowledge. This disappointing finding may be partially attributable to a low capacity of health care supply and a chronic inadequateness of public health care services among village clinics. Village clinics are subject to a lack of health infrastructure, and low educational levels of their health faculty.

It is reported that nearly 90% of village physicians are below vocational educational level, and one third of them practice without a degree [28]. Most of them lack knowledge about CD prevention and control, so it is difficult for them to provide health knowledge to patients. Moreover, most village clinics set up by village or township hospitals are operated as small businesses, although the actual facilities may be owned by the government [29]. In rural areas, nearly 36% of village clinics are privately run and do not offer public health services to rural residents for two main reasons [12]. First is the lack of relevant professional training and equipment available to these practitioners, and the influence this has on the overall quality of health services they provide. The second reason is that a lack of government funding discourages them from paying adequate attention to public health services such as health education and promotion [30].

County hospitals are the centres of medical and technical guidance and training for a county, yet seem to provide less effective health education services than township hospitals. This disparity may be attributable to the heavy burden of diagnosis and treatment of diseases at county hospitals. It was reported in "An Analysis Report of National Health Services Survey in China 2008" that clinicians mainly focused on curing diseases and reversing the unhealthy status of patients, instead of supplying public health care services [31]. In county hospitals, most clinicians are specialists with a high responsibility for managing difficult diseases, and this consumes most of their attention. Therefore, there is no effective coordination of health education, disease prevention and control, and disease therapy for patients [31,32]. Furthermore, because of the profit-oriented nature of county hospitals, physicians pay more attention to disease therapy, which can bring direct economic benefits than to public health care services, and this further contributes to the chronic inadequacy of health education and promotion among county hospitals [29,33].

## Conclusions

Our study highlights the continuing need for greater CD knowledge in the rural areas of China, as well as the positive effects of health care institutions' health education and promotion for rural residents. In addition, our results emphasize the importance of establishing a community based CD prevention and control programme. This would require township hospitals to take a leading role and supply service guidance, technical training, and health supervision to village clinics to increase the quality of public health care services in rural China. Furthermore, efforts should be made to strengthen the basic construction of village clinics, and to develop a team of grass-roots health care workers with the purpose of enhancing health care service capacity in rural areas. Sound supervision of the public health care service system should be established to ensure proper implementation of public health services in rural health institutions.

The sample in this study consists of rural adults with hypertension or T2D, which may or may not represent the chronically ill population. Our data of health knowledge and self-care instructions were based on self-report, which may be subject to respondent bias. The factors influencing attendance at particular hospitals for rural residents with CDs are complicated, such as distance, economic condition, attitude of physicians, diagnosing and treating environment, qualification of the physicians, and waiting times. Our findings may be influenced by these factors, and we assume that distance and economic conditions are likely to be the main factors of influence for participants in Shanxi Province. Another potential limitation of our research is the use of closed-ended questions (i.e. true/false/don't know) in the health knowledge test, which may have allowed participants to guess the correct answer.

## List of abbreviations

CDs: chronic diseases; T2D: type II diabetes; NAHPF: National Health Promotion Project for Hundreds of Millions of Chinese Farmers.

## Competing interests

The authors declare that they have no competing interests.

## Authors' contributions

Study concept and design: MT and YC. Data collection: RZ, LC, XC and DF. Statistical analysis: MT, YC and RZ. Analysis and interpretation of data: MT, RZ and XC. Draft of the manuscript: MT and YC. Critical revision of the manuscript for important intellectual content: CF, MT, YC and LC. Administrative, technical, and material support: CF and MT. Study supervision: CF. All authors read and approved the final manuscript.

## Pre-publication history

The pre-publication history for this paper can be accessed here:

http://www.biomedcentral.com/1471-2458/11/948/prepub

## Supplementary Material

Additional file 1 **Part A (Basic personal information), Part B (Health knowledge of chronic diseases) consulted the question pool of the National Health Promotion Project for Hundreds of Millions of Chinese Farmers (NAHPF) and Part C (The ways of receiving health knowledge) of the questionnaire consulted the questionnaires of the Fourth Chinese National Health Services Survey**. Click here for file
