# Supplementary material for: Chronic disease knowledge and its determinants among chronically ill adults in rural areas of Shanxi Province in China: a cross-sectional study
Source: BMC Public Health. 2011 Dec 22;11:948. doi: 10.1186/1471-2458-11-948 (PMC3268774; doi:10.1186/1471-2458-11-948)
Supplement: Additional file 1 — Part A (Basic personal information), Part B (Health knowledge of chronic diseases) consulted the question pool of the National Health Promotion Project for Hundreds of Millions of Chinese Farmers (NAHPF) and Part C (The ways of receiving health knowledge) of the questionnaire consulted the questionnaires of the Fourth Chinese National Health Services Survey. [file 1471-2458-11-948-S1.DOC]

# Additional files

### Additional file 1 –Questionnaire for patients with chronic disease in rural China

| **Part A Basic personal information** | | **Answer** |
| --- | --- | --- |
| 1 | Date：______________ |  |
| 2 | Whether the questions are answered by yourself ?  (1) Answered by myself (2) Answered by others |  |
| 3 | What kind of chronic diseases are you suffering from?  (1) hypertension (2) type 2 diabetes |  |
| 4 | Your gender：(1)Male (2)Female |  |
| 5 | Your age(year) : ______________ |  |
| 6 | Marital Status：(1)Unmarried (2)Married　(3)Devoiced　(4)widowed |  |
| 7 | Education level：(1) less than 6 years elementary study (2)Primary school  (3) Middle school　(4) beyond high school |  |
| 8 | Occupation：(1)farmer (2)migrant worker (3)self-employed (4)Retired at home (5)Other occupations:___________（Please write down by yourself） |  |
| 9 | How many years have you suffered from the hypertension or diabetes? (length of illness) |  |
| 10 | Whether have any directly related members of your family had your chronic disease? (1)If yes ，he or she is your___________(relationship); (2) no |  |
| 11 | Whether is your condition stable now?  (1) very stable (2) stable (3)a little stable (4)not stable (5)extremely not stable |  |
| **Part B Health knowledge of chronic disease** | |  |
| ***Written by patients with hypertension*** | |  |
| 1 | Do you know the normal blood pressure range of adults?  (1)yes ,I know, it is 　　　mmHg/　　mmHg (2)No , I don’t know |  |
| 2 | **Risk factors relating to hypertension** |  |
| 2.1 | Fat or overweight is one of the risk factor of hypertension.  (1) true; (2)false; (3) I don’t know |  |
| 2.2 | Unhealthy diet habit (such as drinking too much, high salt diet) is one of the risk factor of hypertension.  (1) true; (2)false; (3) I don’t know |  |
| 2.3 | Family history of hypertension is one of the risk factor of hypertension.  (1) true; (2)false; (3) I don’t know |  |
| 2.4 | Hyperglycaemia is one of the risk factor of hypertension.  (1) true; (2)false; (3) I don’t know |  |
| 2.5 | Aging is one of the risk factor of hypertension.  (1) true; (2)false; (3) I don’t know |  |
| 2.6 | Lack of physical activities is one of the risk factor of hypertension.  (1) true; (2)false; (3) I don’t know |  |
| 3 | **Daily self-care** |  |
| 3.1 | Take medicine following the doctor's advice is one effect way to control blood pressure.  (1) true; (2)false; (3) I don’t know |  |
| 3.2 | Having reasonable diet is one effect way to do daily self-care.  (1) true; (2)false; (3) I don’t know |  |
| 3.3 | Maintain good mood is one effect way to do daily self-care.  (1) true; (2)false; (3) I don’t know |  |
| 3.4 | Do appropriate exercise is one effect way to do daily self-care.  (1) true; (2)false; (3) I don’t know |  |
| 3.5 | Control weight is one effect way to do daily self-care.  (1) true; (2)false; (3) I don’t know |  |
| - ***Written by patients with type 2 diabetes*** | |  |
| 1 | Do you know the normal blood glucose of adults?  (1)Yes, I know ,it is___________ mmol/L (2)No , I don’t know |  |
| 2 | **Risk factors relating to diabetes** |  |
| 2.1 | Fat or overweight is one of the risk factor of diabetes.  (1) true; (2)false; (3) I don’t know |  |
| 2.2 | Unhealthy diet habit (such as drinking too much, prefer to eating sweet and oily food) is one of the risk factor of diabetes.  (1) true; (2)false; (3) I don’t know |  |
| 2.3 | Family history of diabetes is one of the risk factor of diabetes.  (1) true; (2)false; (3) I don’t know |  |
| 2.4 | Hypertension is one of the risk factor of diabetes.  (1) true; (2)false; (3) I don’t know |  |
| 2.5 | Aging is one of the risk factor of diabetes.  (1) true; (2)false; (3) I don’t know |  |
| 2.6 | Lack of physical activities is one of the risk factor of diabetes.  (1) true; (2)false; (3) I don’t know |  |
| 3 | **Daily self-care** |  |
| 3.1 | Take medicine under the doctor's advice is one effect way to control blood sugar.  (1) true; (2)false; (3) I don’t know |  |
| 3.2 | Having reasonable diet is one effect way to do daily self-care.  (1) true; (2)false; (3) I don’t know |  |
| 3.3 | Maintain good mood is one effect way to do daily self-care.  (1) true; (2)false; (3) I don’t know |  |
| 3.4 | Do appropriate exercise is one effect way to do daily self-care.  (1) true; (2)false; (3) I don’t know |  |
| 3.5 | Control weight is one effect way to do daily self-care.  (1) true; (2)false; (3) I don’t know |  |
| Part C The ways of receiving health knowledge | |  |
| 1 | Where do you get the above knowledge about chronic disease？**（Please choose the ways you receiving knowledge usually，and not more than 3 items）**  (1)From doctors (2)From family members (3) From friends  (4)From medical books (5)From magazine or newspapers  (6)From TV programs (7)From broadcast  (8)Other ways: （Please write down） |  |
| 2 | Do you want to tell other people about knowledge of CD that you know?  (1)Yes (continue to answer question 2.1); (2)No (skip to question 3) |  |
| 2.1 | Whom do you want to tell about the CD knowledge?  (1)family members (2)neighbours (3)children  (4) other patients with CD (5) all people |  |
| 3 | Which health care institution is your fixed health institution to have regular checkups：  (1)Village clinics (2)Township hospitals (3)County hospitals (4)None |  |
| 4 | Did you get the chronic disease knowledge from the doctors in your fixed health institution？(If the answer is yes, please continue answer the following questions; If the answer is no, please end this part.)  (1)Yes; (2)No |  |
| 5 | Did the doctors tell you that you should measure your blood pressure or blood sugar regularly?  (1)Yes, how often? （Please write down） (2)No |  |
| 6 | Did the doctors tell you that you should stop smoking after you were diagnosed with CD？  (1)Yes; (2)No |  |
| 7 | Did the doctors tell you that you should control your weight after you were diagnosed with CD？  (1)Yes; (2)No |  |
| 8 | Did the doctors tell you that you should often do physical exercises after you were diagnosed CD？  (1)Yes; (2)No |  |
| 9 | Which health institution is the nearest from your home?  (1) Village clinics (2)Private clinics (3)Township hospitals (4)County hospitals  (5)Other institutions: (Please write down) |  |
| 10 | How far is the nearest institution from your home (KM) ? |  |
| 11 | How many minutes does it take you to the fixed institution on foot? |  |
